# Eltrombopag versus romiplostim in treatment of adult patients with immune thrombocytopenia: A systematic review incorporating an indirect-comparison meta-analysis

**DOI:** 10.1371/journal.pone.0198504

**Published:** 2018-06-01

**Authors:** Jiaxing Zhang, Yi Liang, Yuan Ai, Xiaosi Li, Juan Xie, Youping Li, Wenyi Zheng, Rui He

**Affiliations:** 1 Department of Pharmacy, Guizhou Provincial People’s Hospital, Guiyang, China; 2 Chinese Evidence-based Medicine Center, Sichuan University, Chengdu, China; 3 Health Outcomes and Pharmacy Practice, College of Pharmacy, the University of Texas at Austin, Austin, Texas, United States; 4 Department of Pediatric Hematology and Oncology, West China Second University Hospital, Sichuan University, Chengdu, China; 5 Department of Pharmacy, Hospital of Chengdu Office of People’s Government of Tibetan Autonomous Region, Chengdu, China; 6 Experimental Cancer Medicine, Clinical Research Center, Department of Laboratory Medicine, Karolinska Institute, Huddinge, 14186 Stockholm, Sweden; Centro Cardiologico Monzino, ITALY

## Abstract

**Purpose:**

In absence of direct comparison randomized controlled trials (RCTs), indirect comparison was conducted to evaluate the efficacy and safety of thrombopoietin-receptor agonists (TPO-RAs) in treatment of adult immune thrombocytopenia (ITP).

**Methods:**

We searched PubMed, Embase and Cochrane Library, Clinical Trials.gov, China National Knowledge Infrastructure, and Chinese Biomedical Literature Database from their earliest records to May 2017. RCTs comparing the TPO-RAs with placebo in adult ITP were included. Primary outcomes were the overall response rate. Secondary outcomes included safety, durable response, overall or clinically significant bleeding, and the proportion of patients receiving rescue medication.

**Results:**

Nine randomized placebo-controlled trials (786 participants) were included in this systematic review. Overall response [*Risk Ratio(RR)* = 0.59, 95%*Confidence Interval(CI)*: 0.24–1.45], the incidence of adverse events (*RR* = 0.98, 95%*CI*: 0.79–1.21), durable response (*RR* = 0.47, 95%*CI*: 0.08–2.81), the incidence of overall bleeding (*RR =* 1.15, 95%*CI*: 0.52–2.57) and clinically significant bleeding (*RR =* 1.09, 95%*CI*: 0.37–3.24), and the proportion of patients receiving rescue treatment (*RR* = 0.95, 95%*CI*: 0.47–1.90) were similar between eltrombopag and romiplostim.

**Conclusions:**

Eltrombopag and romiplostim might be equivalent in efficacy and safety for adult ITP, however, physicians should still take into account drug cost and comorbidities of the specific patient while making decisions on the treatment of ITP with TPO-RAs.

**Registration:**

PROSPERO International Prospective Register of Systematic Review (PROSPERO 2017: CRD42017068661).

## Introduction

Immune thrombocytopenia (ITP) is an immune-mediated disease characterized by transient or persistent decrease in the platelet count and increased risk of bleeding [1]. ITP in adults is a clinically distinct condition from that in children, with a lower likelihood of spontaneous remission, a higher incidence of underlying diseases and comorbidities, and often a higher risk of bleeding. The incidences of adult ITP reported in recent studies varied among countries: 2.20 per 10^5^ in Japan [2], 2.94 per 10^5^ in France [3], and 3.70 per 10^5^ in Korea [4], respectively. ITP is often a chronic disease in adults, and the prevalence exceeds the incidence [5].

The first-line treatments of adult ITP include longer courses of corticosteroids, intravenous immunoglobulin (IVIg) and anti-D immunoglobulin which has been approved for ITP only in a few countries (North America) [6]. But for those refractory to the first-line treatments, subsequent treatment may include splenectomy, rituximab, thrombopoietin-receptor agonists (TPO-RAs), or more potent immunosuppression [6]. TPO-RAs stimulating the TPO-receptor to increase the production of platelets are recommended for adults at risk of bleeding who relapse after splenectomy, or who have a contraindication to splenectomy and who have failed at least one other therapy [6]. Two TPO-RAs, eltrombopag (ELT) and romiplostim (ROM), have been approved for the treatment of adults with ITP in the United States. Recent evidence showed that TPO-RAs were effective and safe second-line options for primary ITP patients [7].

Nevertheless, ROM and ELT have different mechanisms of action and routes of administration: ROM is a subcutaneously administered peptide mimetic binding to the extracellular TPO-receptor, while ELT is an oral non-peptide binding to a transmembrane site of the TPO-receptor [8, 9]. Unfortunately, there are no head-to-head randomized controlled trials (RCTs) comparing ROM with ELT in treatment of adult ITP. Hence indirect comparisons, which preserves within-trial randomization by comparison treatment effects(RR) relative to a common comparator (placebo) from each trial [10], are recommended in the UK National Institute for Health and Clinical Excellence (NICE) methods guide [11]. An indirect comparison between ROM and ELT in treatment of adult patients with ITP was previously conducted [12], but the conclusions became controversial with the publication of later RCTs. Therefore, this study aims to evaluate the efficacy and safety of ELT versus ROM for adult patients with ITP using an indirect-comparison meta-analysis.

## Materials and methods

We followed the standards set by Preferred Reporting Items for Systematic reviews and Meta-Analyses (PRISMA) in this systematic review (S1 Table). The study was registered in PROSPERO International Prospective Register of Systematic Review (PROSPERO 2017: CRD42017068661).

### Literature search

Pubmed, Embase, and Cochrane Central Register of Controlled Trials (CENTRAL) published in Cochran Library were searched using the search strategies detailed in S2 Table, from their earliest records to May 2017. Clinical Trials.gov was searched using the terms “immune thrombocytopenia”, “adult”, “eltrombopag”, and “romiplostim”. The China National Knowledge Infrastructure(CNKI) and Chinese Biomedical Literature Database(CBLD) were also searched in Chinese.

### Eligibility criteria

All included studies met the following criteria: (1) Randomized controlled studies; (2) Participants were adult (≥ 18 years) with ITP; (3) the intervention was ELT or ROM irrespective of dosage and schedule; (4) the comparison was placebo; (5) studies included at least one of the following outcomes: overall platelet response(primary outcome), defined as achieving at least once platelet response (≥ 50×10^9^/L) during treatment; incidence of overall and serious adverse events (SAEs); durable platelet response, defined as maintaining platelet counts ≥ 50×10^9^/L for at least 60% of the duration of TPO-RAs treatment or for six or more weeks during the final eight weeks of TPO-RAs treatment; incidence of clinically significant bleeding (WHO Grade 2–4 or rated as severe, life threatening, or fatal); all bleeding events; and proportion of patients who received rescue treatment [e.g. receiving any unscheduled or new treatment (including new drugs, increase dose of a concomitant drug from baseline, platelet transfusion or splenectomy) for immediate risk or treatment failure]; (6) publications written in English or Chinese. We excluded studies on patients with secondary ITP and those including both children and adults when data of adults could not be extracted separately.

### Study selection and data extraction

Two authors independently screened the titles and abstracts of all studies identified by the search strategies, and assessed the studies using predetermined inclusion criteria. The full texts of all potentially relevant articles were retrieved for detailed review. We resolved any disagreements by discussion until consensus was achieved. We used a pre-designed data collection form to extract data from each eligible study. The following data were extracted: (1) authors; (2) year of publication; (3) country or region where the study conducted; (4) study design and use of control; (5) number of participants randomized into each group; (6) gender, age, and disease duration of participants; (7) baseline platelet count, previous ITP medication, and splenectomy status; (8) dose and schedule of TPO-RAs; (9) outcomes of each study and their definitions; (10) numerical data for assessment of included outcomes; (11) sources of funding.

### Quality assessment

Two authors independently assessed the risk of bias of each included study using the checklist developed by Cochrane Collaboration [13]. The items included random sequence generation, allocation concealment, blinding, incomplete outcome data, selective outcome reporting, and other bias. We categorized the judgments as low, high or unclear risk of bias and created plots of risk of bias assessment in Review Manager Software (RevMan 5.3).

### Statistical synthesis

We calculated a kappa statistic for measuring the agreement level between two authors making decisions on study selection. The value of kappa (*K*) between 0.40 and 0.59 was considered as fair agreement, between 0.60 and 0.74 as good and 0.75 or more as excellent.

If more than one study reported the same outcome, the pairwise meta-analysis was conducted to calculate the pooled estimate of the risk ratio (*RR*) of different TPO-RAs versus placebo by RevMan 5.3. Statistical heterogeneity among studies was examined by the Chi-square test and quantified by the *I*^*2*^ statistic [14]. We used a fixed-effect model to synthesize data when heterogeneity was not significant (*P*>0.1 and *I*^*2*^<50%). When heterogeneity was significant (*P*≤0.1 and *I*^*2*^≥50%) and could not be explained by subgroup analyses or in terms of clinical or methodological features of the trials, the random-effect model was used. If both the ELT-placebo and ROM-placebo trials reported the same outcome, the relative treatment effect (*RR*) for ELT versus ROM was estimated using indirect procedure of Stata12.0 software [15], with the formula as follow:

$$RR_{ELT/ROM}=RR_{ELT/Placebo}/RR_{ROM/Placebo},$$

$$Variance\;\left(log\;RR_{ELT/ROM}\right)=Variance\;\left(log\;RR_{ELT/Placebo}\right)+Variance\;\left(log\;RR_{ROM/Placebo}\right).$$

For the overall platelet response, sensitivity analysis was conducted by comparing the results of intention-to-treat (ITT) analysis with per-protocol (PP) analysis to improve the robustness of the results. The subgroup analysis was also conducted according to the different types of adverse events(AEs).

## Results

### Study selection

**Fig 1:**
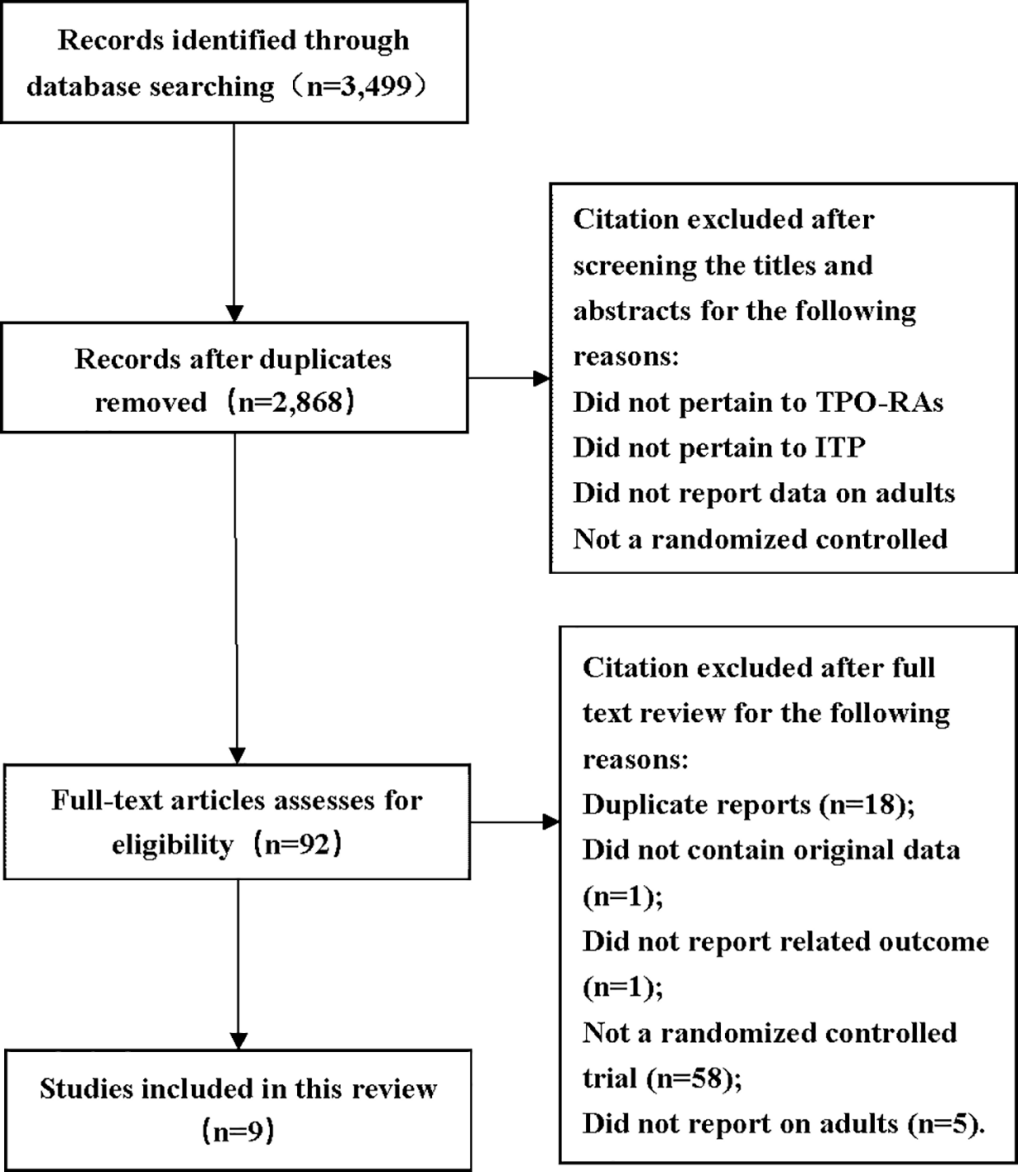
Flow diagram of study selection process for this systematic review.

A total of 3,499 citations were obtained from the literature search and the selection process was shown in Fig 1. Nine randomized, placebo-controlled studies (786 participants) [16–23] were included in this systematic review, and two of them were published in one article [22]. Agreement between two reviewers for study selection was excellent (*K* = 0.85). As shown in Table 1, all studies were multicenter, double-blind, RCTs from different countries in North and South America, Asia, Europe, Africa, and Oceania.

**Table 1:** Characteristics of included studies.

Study ID	Study Design	Population inclusion	Intervention vs Comparison	TPO-RA regimens	Outcomes
Bussel 2007 [16]	Multicenter (44 centers in 14 countries), double-blind, RCT.	Patients≥18 years old, with a diagnosis of ITP(duration of ≥6 months), had received at least one previous treatment for ITP, and had a platelet counts<30×10^9^/L.	Eltrombopag vs Placebo	30, 50, or 75mg orally daily for 6 weeks.	①④⑥⑦
Bussel 2009 [17]	Multicenter (63 centers in 23 countries), double-blind, RCT.	Patients≥18 years old, with a diagnosis of ITP(duration of ≥6 months), had received at least one previous treatment for ITP, and had a platelet counts<30×10^9^/L.	Eltrombopag vs Placebo	50mg orally daily for 6 weeks; dose was adjusted based on platelet counts.	①④⑥⑦
Cheng 2011 [18]	Multicenter (75 centers in 23 countries), double-blind, RCT.	Patients≥18 years old, with a diagnosis of ITP(duration of ≥6 months), had received at least one previous treatment for ITP, and had a platelet counts<30×10^9^/L.	Eltrombopag vs Placebo	50mg orally daily for 24 weeks; dose was adjusted based on platelet counts.	①②③④⑤⑥⑦
Tomiyama 2012 [19]	Multicenter (7 centers in Japan), double-blind, RCT.	Patients≥20 years old, with a diagnosis of ITP(duration of ≥6 months), had received at least one previous treatment for ITP, and had a platelet counts<30×10^9^/L.	Eltrombopag vs Placebo	Starting dose of 12.5mg (maximum dose of 50mg) orally daily for 6 weeks; dose was adjusted based on platelet counts.	①②⑥⑦
Yang 2017 [20]	Multicenter (16 centers in China), double-blind, RCT.	Patients≥18 years old, with a diagnosis of ITP(duration of ≥12 months), had received at least one previous treatment for ITP, had a platelet counts<30×10^9^/L.	Eltrombopag vs Placebo	25 mg once daily for 8 weeks; dose was adjusted based on platelet counts.	①②③④⑤⑥⑦
Bussel 2006 [21]	Multicenter (9 centers in USA), double-blind, RCT.	Patients(18–65 years old), with a diagnosis of ITP(duration of ≥3 months), had received at least one previous treatment for ITP, and had a platelet counts<30×10^9^/L.	Romiplostim vs Placebo	1 or 3ug/kg subcutaneously weekly for 6 weeks, 8 patients with 1ug/kg, 8patients with 3ug/kg, 1 patients with 6ug/kg, no dose adjustments	①⑦
Kuter 2008a [22]	Multicenter (35 centers in the USA and Europe), double-blind, RCT.	Patients≥18 years old, with a diagnosis of ITP(duration of ≥6 months), had received at least one previous treatment for ITP, had a platelet counts<30×10^9^/L, and had a splenectomy for the treatment of ITP greater than or equal to 24 weeks prior to study entry.	Romiplostim vs Placebo	Starting dose of 1ug/kg subcutaneously weekly for 24 weeks; dose was adjusted to achieve target platelet counts of 50 to 200×10^9^/L.	①②③④⑤⑥⑦
Kuter 2008b [22]	Multicenter (35 centers in the USA and Europe), double-blind, RCT.	Patients≥18 years old, with a diagnosis of ITP(duration of ≥6 months), had received at least one previous treatment for ITP, had a platelet counts<30×10^9^/L, and had non-splenectomized status.	Romiplostim vs Placebo	Starting dose of 1ug/kg subcutaneously weekly for 24 weeks; dose was adjusted to achieve target platelet counts of 50 to 200×10^9^/L.	①②③④⑤⑥⑦
Shirasugi 2011 [23]	Multicenter (11 centers in Japan), double-blind, RCT.	Patients≥20 years old, with a diagnosis of ITP(duration of ≥6 months), had received at least one previous treatment for ITP, and had a platelet counts<30×10^9^/L.	Romiplostim vs Placebo	starting dose of 3ug/kg subcutaneously weekly for 12 weeks; dose was adjusted to achieve target platelet counts of 50 to 200×10^9^/L.	③④⑤⑥⑦

①Platelet response

②Durable platelet response

③Clinically significant bleeding

④All bleeding events

⑤Rescue medication

⑥Adverse events

⑦Serious adverse events.

### Description of patients

All patients were aged ≥18 years old, with disease duration more than 3 months and baseline platelet count less than 30×10^9^/L. Five studies (606 patients) evaluated the efficacy and safety of ELT in comparison to placebo [16–20] (Table 1). The initial dose of ELT was ranged from 12.5 to 75mg, and the following dose was adjusted according to individual platelet count, with a target of 50–200×10^9^/L [16]. Four studies (180 patients) evaluated the efficacy and safety of ROM [21–23]. It was administrated at an initial dose of 1 or 3 μg/kg and was also adjusted according to platelet counts (Table 1). The characteristics of patients were shown in Table 2.

**Table 2:** Characteristics of included patients.

Study ID	Participants(n): TPO-RA vs Control	Gender: Female/Male(n): TPO-RA vs Control	Age(years): TPO-RA vs Control	Duration of ITP(years): TPO-RA vs Control	Splenectomy status(yes/no)(n): TPO-RA vs Control	Baseline platelet count(10^9^/L): TPO-RA vs Control	Concomitant ITP medication: TPO-RA vs Control
Bussel 2007 [16]	88(ELT) vs 29(PLA)	57/31 vs 16/13	51(23–79);45(23–81);55(18–85) vs 42(18–85)	>0.5 vs >0.5	41/47 vs 14/15	PC ≤15×10^9^/L: 42/88 vs 14/29	32/88 vs 6/29
Bussel 2009 [17]	76(ELT) vs 38(PLA)	43/33 vs 27/11	47(19–84) vs 51(21–79) 51±17 vs 48±16	>0.5 vs >0.5	31/45 vs 14/24	PC ≤15×10^9^/L: 38/76 vs 17/38	32/76 vs 17/38
Cheng 2011 [18]	135(ELT) vs 62(PLA)	93/42 vs 43/19	47.0(34–56) vs 52.5(43–63)	>0.5 vs >0.5	50/85 vs 21/41	16(8–22) vs 16(9–24) PC ≤15×10^9^/L: 67/135 vs 30/62	63/135 vs 31/62
Tomiyama 2012 [19]	15(ELT) vs 8(PLA)	8/7 vs 7/1	58.0(26–72) vs 60.5(38–72)	>0.5 vs >0.5	11/4 vs 5/3	21(16–25) vs 9.5(7.5–19) PC ≤15×10^9^/L: 3/15 vs 6/8	12/15 vs 7/8
Yang 2017 [20]	104(ELT) vs 51(PLA)	77/27 vs 40/11	48(18–84) vs 42(22–66) 44.7 ±15.91 vs 41.3±12.83	>1.0 vs >1.0	18/86 vs 7/44	14.0 vs 13.5 PC ≤15×10^9^/L: 54/104 vs 28/51	53/104 vs 28/51
Bussel 2006 [21]	17(ROM) vs 4(PLA)	12/5 vs 3/1	45(20–63);53(19–62);42 vs 55(39–64)	5.6(0.5–24.9);9.1(0.4–37.0); 6.4 vs 3.4(0.8–3.7)	13/4 vs 1/3	17(4–25);12(5–23);15 vs 29(6–49)	4/17 vs 3/4
Kuter 2008a [22]	42(ROM) vs 21(PLA)	27/15 vs 11/10	51(27–88) vs 56(26–72)	7.8(0.6–44.8) vs 8.5(1.1–31.4)	42/0 vs 21/0	14(3–29) vs 15(2–28)	12/42 vs 6/21
Kuter 2008b [22]	41(ROM) vs 21(PLA)	27/14 vs 16/5	52(21–80) vs 46(23–88)	2.2(0.1–31.6) vs 1.6(0.1–16.2)	0/41 vs 0/21	19(2–29) vs 19(5–31)	11/41 vs 10/21
Shirasugi 2011 [23]	22(ROM) vs 12(PLA)	14/8 vs 10/2	58.5±12.6 vs 47.6±13.4	9.7±10.4 vs 7.6±5.9	10/12 vs 5/7	18.4±8.3 vs 15.8±6	13/22 vs 10/12

PC: Platelet count; ELT: Eltrombopag; ROM: Romiplostim; PLA: Placebo.

### Quality assessment

**Fig 2:**
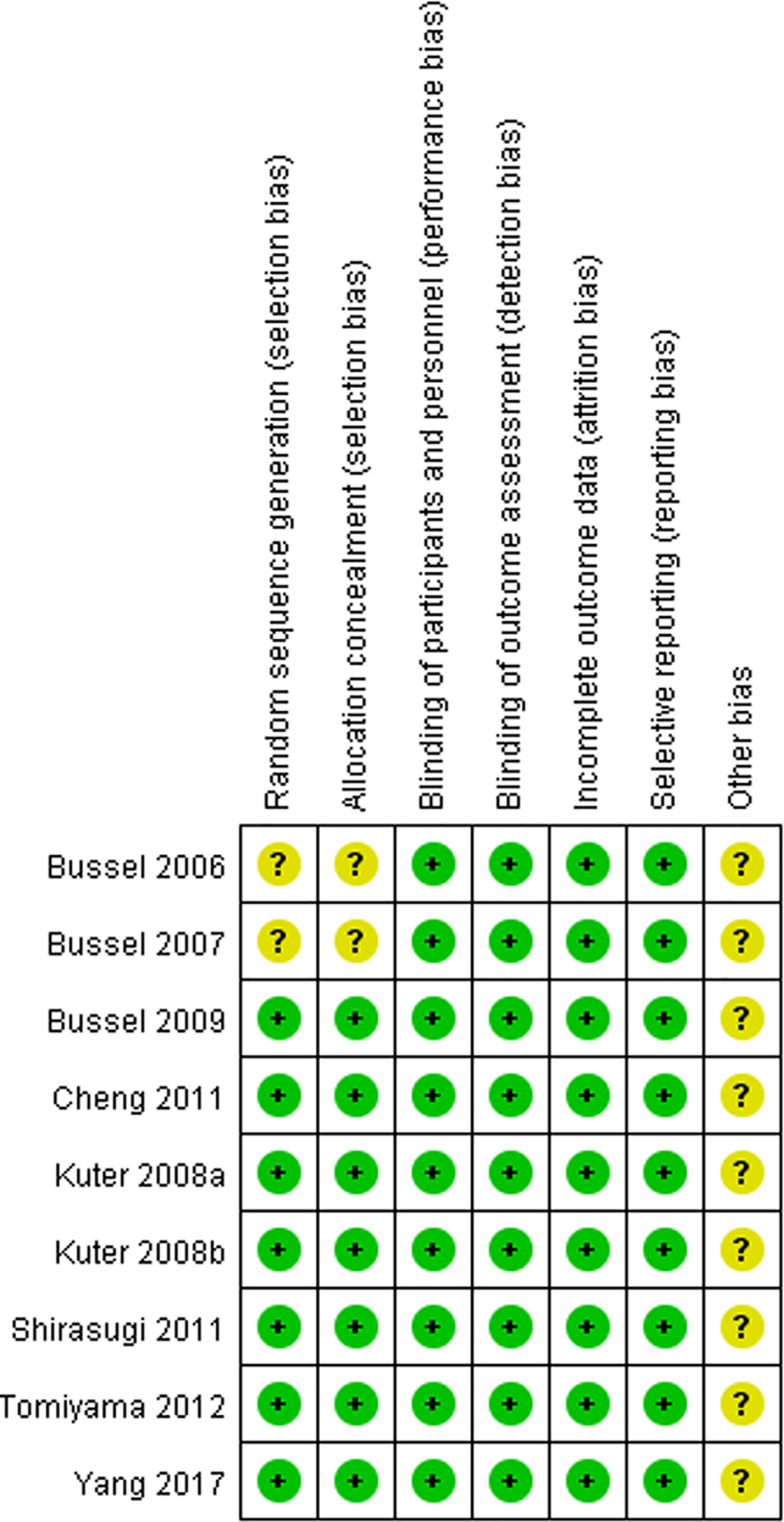
Risk of bias summary.

As shown in Fig 2, seven studies [17–20, 22, 23] had low risk of selection bias for central randomization while the other two was unclear because the method of randomization and allocation concealment were not reported [16, 21]. All studies [16–23] had low risk of performance bias and detection bias, as both patients and study personnel were masked. All studies [16–23] had low risk of attrition bias, as there was no loss to follow-up or the missing data were dealt with properly (e.g. applying ITT analysis which underestimated the efficacy of the medication). All studies [16–23] had low risk of reporting bias since they were registered in *ClinicalTrials*.*gov* and had reported all predesigned outcomes. Considering all studies [16–23] supported by pharmaceutical industry, the bias caused by conflict of interest was unclear.

### Overall platelet response

**Fig 3:**
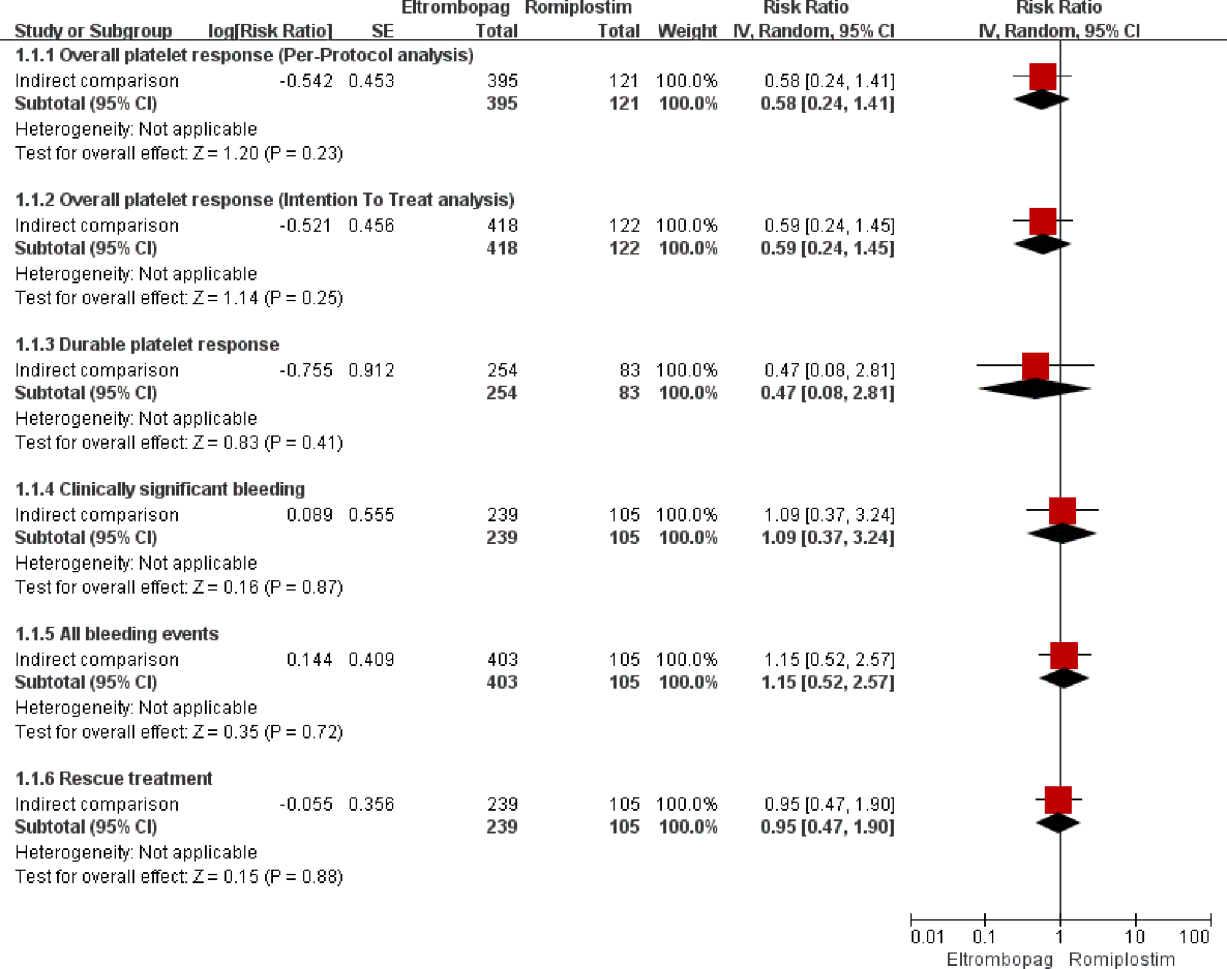
The efficacy results of indirect-comparison meta-analysis.

The overall platelet response was reported in all studies (five for ELT [16–20] and four for ROM [21–23], respectively) including 785 patients (ITT). The heterogeneity was not statistically significant (*I*^*2*^ = 32%, *P* = 0.21 and *I*^*2*^ = 4%, *P* = 0.37, respectively). The pooled results with a fixed-effect model (Table 3) showed that proportion of patients achieving overall response was significantly higher in the TPO-RAs group than in the placebo group (*RR* = 4.07, 95%*CI*: 2.91–5.70 for ELT and *RR* = 8.81, 95%*CI*: 4.01–19.35 for ROM, respectively). However, the result of indirect comparison (Fig 3) indicated that the overall response between ELT and ROM was not significantly different (*RR* = 0.59, 95%*CI*: 0.24–1.45). And sensitivity analysis showed that the results of PP analysis were consistent with the ITT analysis (Table 3 and Fig 3).

**Table 3:** The direct comparison meta-analysis results of outcomes.

Outcomes	TPO-RA vs PLA	n	N (TPO-RA vs PLA)	Heterogeneity	Model	*RR*	*95%CI*	*P*
**Overall platelet response(PP)**	ELT vs PLA	5	395 vs 179	*I*^*2*^ = 29%, *P* = 0.23	Fixed	4.05	[2.90, 5.66]	<0.00001
	ROM vs PLA	4	121 vs 58	*I*^*2*^ = 0%, *P* = 0.39	Fixed	8.86	[4.03, 19.48]	<0.00001
**Overall platelet response(ITT)**	ELT vs PLA	5	418 vs 187	*I*^*2*^ = 32%, *P* = 0.21	Fixed	4.07	[2.91, 5.70]	<0.00001
	ROM vs PLA	4	122 vs 58	*I*^*2*^ = 4%, *P* = 0.37	Fixed	8.81	[4.01, 19.35]	<0.00001
**Durable platelet response**	ELT vs PLA	3	254 vs 120	*I*^*2*^ = 0%, *P* = 0.85	Fixed	6.82	[2.97, 15.70]	<0.00001
	ROM vs PLA	2	83 vs 42	*I*^*2*^ = 0%, *P* = 0.87	Fixed	14.16	[2.91, 69.01]	0.001
**Clinically significant bleeding**	ELT vs PLA	2	239 vs 112	*I*^*2*^ = 0%, *P* = 0.83	Fixed	0.64	[0.46, 0.90]	0.009
	ROM vs PLA	3	105 vs 54	*I*^*2*^ = 0%, *P* = 0.87	Fixed	0.43	[0.14, 1.33]	0.14
**All bleeding events**	ELT vs PLA	4	403 vs 179	*I*^*2*^ = 53%, *P* = 0.09	Random	0.76	[0.60, 0.97]	0.03
	ROM vs PLA	3	105 vs 54	*I*^*2*^ = 81%, *P* = 0.02	Random	0.68	[0.31, 1.48]	0.33
**Rescue medication**	ELT vs PLA	2	239 vs 112	*I*^*2*^ = 35%, *P* = 0.22	Fixed	0.37	[0.25, 0.54]	<0.00001
	ROM vs PLA	3	105 vs 54	*I*^*2*^ = 0%, *P* = 0.55	Fixed	0.38	[0.24, 0.60]	<0.0001
**All adverse events**	ELT vs PLA	5	418 vs 187	*I*^*2*^ = 63%, *P* = 0.03	Random	1.05	[0.84, 1.32]	0.68
	ROM vs PLA	3	106 vs 53	*I*^*2*^ = 0%, *P* = 0.84	Fixed	1.05	[0.97, 1.14]	0.26
**Serious adverse events**	ELT vs PLA	5	418 vs 187	*I*^*2*^ = 0%, *P* = 0.73	Fixed	0.93	[0.54, 1.59]	0.79
	ROM vs PLA	4	123 vs 57	*I*^*2*^ = 11%, *P* = 0.34	Fixed	0.77	[0.46, 1.29]	0.32
**Headache**	ELT vs PLA	5	418 vs 187	*I*^*2*^ = 0%, *P* = 0.86	Fixed	0.89	[0.61, 1.28]	0.53
	ROM vs PLA	4	122 vs 58	*I*^*2*^ = 0%, *P* = 0.63	Fixed	1.30	[0.79, 2.14]	0.30
**Fatigue**	ELT vs PLA	4	314 vs 136	*I*^*2*^ = 35%, *P* = 0.20	Fixed	0.66	[0.35, 1.23]	0.19
	ROM vs PLA	4	122 vs 58	*I*^*2*^ = 0%, *P* = 0.83	Fixed	1.23	[0.71, 2.12]	0.47
**Thrombosis**	ELT vs PLA	4	342 vs 149	*I*^*2*^ = 0%, *P* = 0.96	Fixed	1.83	[0.40, 8.43]	0.44
	ROM vs PLA	3	100 vs 46	*I*^*2*^ = 32%, *P* = 0.22	Fixed	0.42	[0.09, 2.11]	0.30
**Arthralgia**	ELT vs PLA	3	299 vs 128	*I*^*2*^ = 47%, *P* = 0.15	Fixed	0.74	[0.31, 1.81]	0.52
	ROM vs PLA	3	100 vs 46	*I*^*2*^ = 65%, *P* = 0.09	Random	0.55	[0.04, 6.86]	0.64
**Nausea**	ELT vs PLA	3	226 vs 107	*I*^*2*^ = 0%, *P* = 0.69	Fixed	2.26	[0.89, 5.74]	0.09
	ROM vs PLA	3	100 vs 46	*I*^*2*^ = 0%, *P* = 0.37	Fixed	1.18	[0.45, 3.06]	0.74
**Nasopharyngitis**	ELT vs PLA	3	226 vs 107	*I*^*2*^ = 0%, *P* = 0.44	Fixed	0.98	[0.50, 1.89]	0.94
	ROM vs PLA	3	105 vs 54	*I*^*2*^ = 71%, *P* = 0.06	Random	1.04	[0.22, 4.87]	0.96
**Diarrhea**	ELT vs PLA	3	299 vs 128	*I*^*2*^ = 31%, *P* = 0.24	Fixed	1.09	[0.52, 2.26]	0.82
	ROM vs PLA	2	83 vs 42	NA	NA	1.18	[0.49, 2.85]	0.71
**Peripheral edema**	ELT vs PLA	2	223 vs 90	*I*^*2*^ = 0%, *P* = 0.53	Fixed	0.20	[0.06, 0.66]	0.008
	ROM vs PLA	2	39 vs 16	*I*^*2*^ = 0%, *P* = 0.38	Fixed	2.75	[0.38, 19.65]	0.31
**Epistaxis**	ELT vs PLA	2	223 vs 90	*I*^*2*^ = 24%, *P* = 0.25	Fixed	0.74	[0.28, 1.90]	0.52
	ROM vs PLA	3	100 vs 46	*I*^*2*^ = 0%, *P* = 0.44	Fixed	1.26	[0.73, 2.19]	0.41
**Pain in extremity**	ELT vs PLA	2	164 vs 67	*I*^*2*^ = 0%, *P* = 0.50	Fixed	0.38	[0.06, 2.31]	0.29
	ROM vs PLA	3	105 vs 54	*I*^*2*^ = 0%, *P* = 0.83	Fixed	3.01	[0.82, 11.05]	0.10
**Dizziness**	ELT vs PLA	2	211 vs 99	*I*^*2*^ = 0%, *P* = 0.64	Fixed	0.34	[0.12, 0.98]	0.05
	ROM vs PLA	3	100 vs 46	*I*^*2*^ = 80%, *P* = 0.02	Random	2.42	[0.05, 126.08]	0.66
**Contusion**	ELT vs PLA	1	135 vs 61	NA	NA	0.3	[0.05, 1.76]	0.18
	ROM vs PLA	4	122 vs 58	*I*^*2*^ = 12%, *P* = 0.32	Fixed	0.86	[0.52, 1.42]	0.55
**Abdominal pain upper**	ELT vs PLA	2	211 vs 99	*I*^*2*^ = 0%, *P* = 0.96	Fixed	0.54	[0.19, 1.54]	0.25
	ROM vs PLA	2	83 vs 42	NA	NA	9.73	[0.58, 163.17]	0.11
**Upper respiratory tract infection**	ELT vs PLA	2	211 vs 99	*I*^*2*^ = 0%, *P* = 0.52	Fixed	1.01	[0.45, 2.28]	0.98
	ROM vs PLA	2	83 vs 42	NA	NA	1.42	[0.55, 3.67]	0.47
**Cough**	ELT vs PLA	2	211 vs 99	*I*^*2*^ = 0%, *P* = 0.42	Fixed	0.54	[0.18, 1.65]	0.28
	ROM vs PLA	2	83 vs 42	NA	NA	0.72	[0.30, 1.76]	0.48
**Myalgia**	ELT vs PLA	2	211 vs 99	*I*^*2*^ = 0%, *P* = 0.69	Fixed	2.14	[0.56, 8.21]	0.27
	ROM vs PLA	2	83 vs 42	NA	NA	6.07	[0.82, 45.14]	0.08
**Anxiety**	ELT vs PLA	2	211 vs 99	*I*^*2*^ = 0%, *P* = 0.75	Fixed	0.26	[0.06, 1.20]	0.08
	ROM vs PLA	2	83 vs 42	NA	NA	0.91	[0.33, 2.55]	0.86
**Back pain**	ELT vs PLA	1	135 vs 61	NA	NA	1.05	[0.28, 3.94]	0.94
	ROM vs PLA	3	105 vs 54	*I*^*2*^ = 0%, *P* = 0.50	Fixed	1.67	[0.61, 4.55]	0.32

n: number of included studies; N: number of patients; ELT: Eltrombopag; ROM: Romiplostim; PLA: Placebo; *RR*: Risk Ratio; *CI*: confidence interval; NA: not applicable; Fixed: Fixed-effect model; Random: Random-effect model.

### Safety

**Fig 4:**
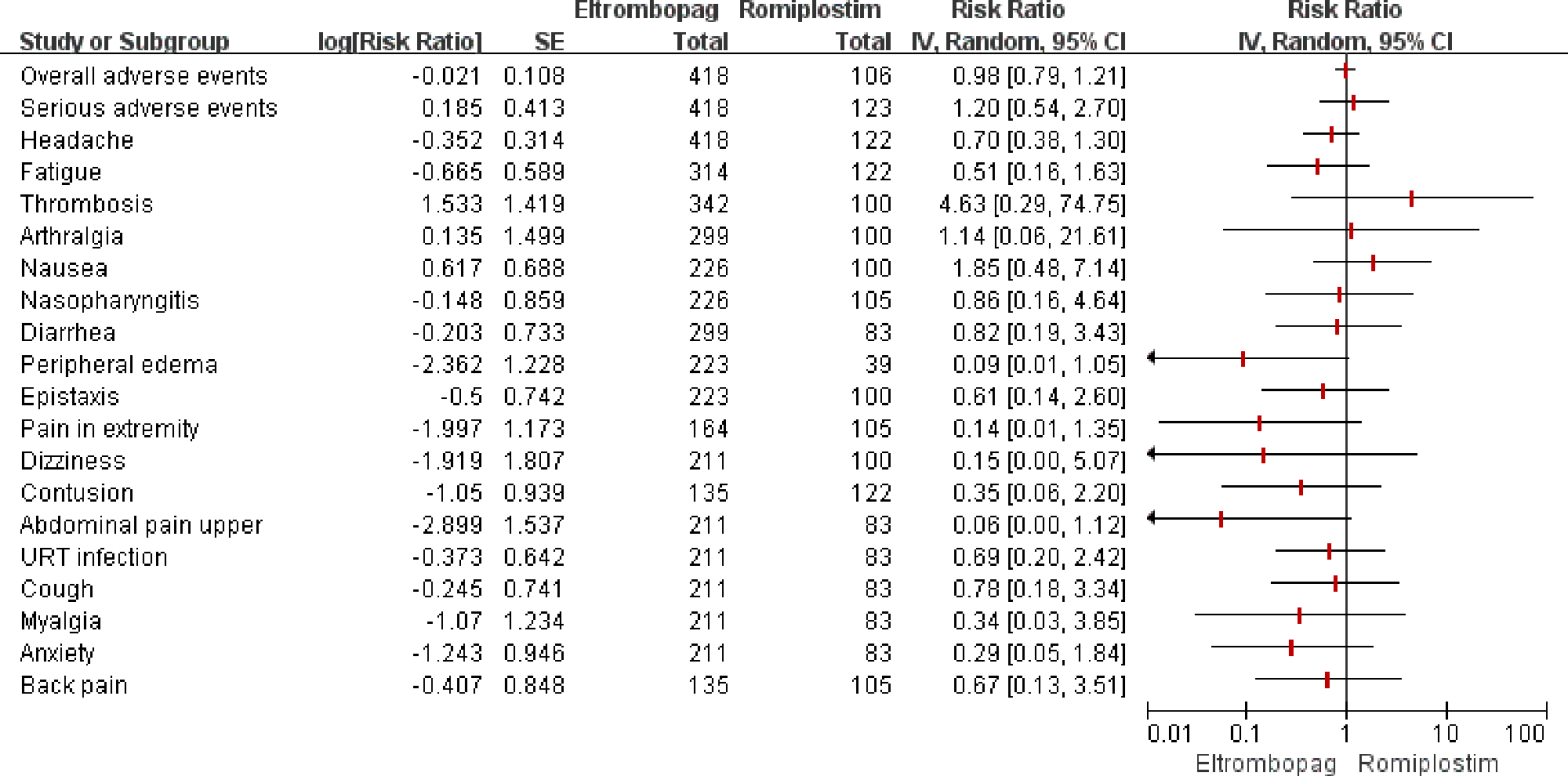
The safety results of indirect-comparison meta-analysis. URT: upper respiratory tract.

Eight studies (764 participants) [16–20, 22–23] reported the overall incidence of any AEs reported in patients receiving TPO-RAs or placebo. The pooled analysis showed that the incidence was not significantly different between two groups (*RR* = 1.05, 95%*CI*: 0.84–1.32 for ELT and *RR* = 1.05, 95%*CI*: 0.97–1.14 for ROM) (Table 3). And the result of indirect comparison (Fig 4) also showed that the overall incidence of any AEs in ELT group was similar to that in ROM group (*RR* = 0.98, 95%*CI*: 0.79–1.21).

SAEs were reported in all studies [16–23], and the results of both direct and indirect comparison (Table 3 and Fig 4) indicated that the incidences of SAEs among ELT, ROM and placebo were not significantly different (ELT vs Placebo: *RR* = 0.93, 95%*CI*: 0.54–1.59; ROM vs Placebo: *RR* = 0.77, 95%*CI*: 0.46–1.29; ELT vs ROM: *RR* = 1.20, 95%*CI*: 0.54–2.70; respectively).

The common AEs in TPO-RAs or placebo group were headache, fatigue, thrombosis, arthralgia, nausea, nasopharyngitis, diarrhea, peripheral edema, epistaxis, pain in extremity, dizziness, contusion, upper abdominal pain, upper respiratory tract infection, cough, myalgia, anxiety and back pain. However, both the direct and indirect comparison of the incidences (Table 3 and Fig 4) demonstrated no significant difference among ELT, ROM and placebo, except that the incidence of peripheral edema was significantly lower in ELT than in placebo (*RR* = 0.20, 95%*CI*: 0.06–0.66). As liver abnormalities and cataract were only reported in ELT-placebo trials, indirect comparison was not conducted. Head to head meta-analysis results indicated that incidences of increased level of ALT or AST and cataract were not significantly different between ELT and placebo (ALT: *RR* = 1.39, 95%*CI* = [0.31, 4.24]; AST: *RR* = 1.37, 95%*CI* = [0.55, 3.40]; Cataract: *RR* = 0.69, *CI* = [0.24, 1.98], respectively). Kuter 2008a reported that a splenectomized, non-responding patient developed an increased reticulin level during ROM treatment, but this level of reticulin subsequently returned to baseline after termination of ROM [22]. Bussel 2006 reported that two patients receiving ROM exhibited reversible increases in bone marrow reticulin levels during the subsequent extension study [21].

### Durable platelet response

Five studies [18–20, 22] reported the durable platelet response, including 499 patients. The direct and indirect comparison analysis (Table 3 and Fig 3) showed that proportion of patients achieving durable platelet response in TPO-RAs was significantly higher than placebo (*RR* = 6.82, 95%*CI*: 2.97–15.70 for ELT and *RR* = 14.16, 95%*CI*: 2.91–69.01 for ROM), but there was no significant difference between ELT and ROM (*RR* = 0.47, 95%*CI*: 0.08–2.81).

### Clinically significant bleeding

Five studies (510 patients) reported the incidence of clinically significant bleeding [18, 20, 22, 23]. According to the direct comparison (Table 3), the incidence was significantly lower in patients receiving ELT than those receiving placebo (*RR* = 0.64, 95%*CI*: 0.46–0.90), but the incidence was not significantly different between ROM and placebo (*RR =* 0.43, 95%*CI*: 0.14–1.33). As to indirect comparison (Fig 3), the incidence was not significantly different between ELT and ROM (*RR =* 1.09, 95%*CI*: 0.37–3.24).

### All bleeding events

Seven studies (741 patients) reported the incidence of all bleeding events [16–18, 20, 22, 23]. The pooled results (Table 3) demonstrated that the incidence was significantly lower in ELT group compared with placebo group (*RR =* 0.76, 95%*CI*: 0.60–0.97), while the incidence was not statistically different between ROM and placebo (*RR =* 0.68, 95%*CI*: 0.31–1.48). According to the indirect comparison (Fig 3), the incidence was not significantly different between ELT and ROM (*RR =* 1.15, 95%*CI*: 0.52–2.57).

### Rescue treatment

Five studies (510 patients) reported the proportion of patients receiving rescue treatment in TPO-RAs or placebo group [18, 20, 22, 23]. Head to head comparison (Table 3) indicated that TPO-RAs could significantly reduce the use of rescue medication compared to placebo (*RR* = 0.37, 95%*CI*: 0.25–0.54 for ELT and *RR* = 0.38, 95%*CI*: 0.24–0.60 for ROM, respectively). However, the indirect comparison (Fig 3) indicated that the proportion of patients receiving rescue treatment between ELT and ROM was not significantly different (*RR* = 0.95, 95%*CI*: 0.47–1.90).

## Discussion

This systematic review incorporating an indirect-comparison meta-analysis summarized the efficacy and safety of TPO-RAs in adults with ITP. Our study suggests that the use of TPO-RAs may improve the durable and overall platelet response, and reduce the use of rescue medication, without increasing the incidence of AEs, compared to placebo. While ELT might resemble ROM in the overall and durable platelet response, the incidence of AEs (including SAEs), the incidence of overall and clinically significant bleeding, and the proportion of patients receiving rescue treatment.

A published indirect comparison demonstrated that ROM significantly improved overall platelet response compared with ELT for adult patients with ITP, while the durable platelet response of the two TPO-RAs was similar [12, 24]. Our research drew a different conclusion that ELT and ROM were similar in both overall and durable platelet response for adults with ITP, as additional studies led to negative results in the indirect comparison analysis.

In addition, an observational retrospective study including 280 adult patients (ELT, n = 130; ROM, n = 150) with chronic ITP concluded that the clinical outcomes between the ELT and ROM treatment cohorts were not significantly different [25]. A “real life” retrospective multicenter study including 124 adult patients (ELT, n = 69; ROM, n = 55) with ITP concluded that the two drugs demonstrated comparable efficacy and risk of thrombotic events (*RR* = 1.59, 95%*CI*: 0.15–17.13) [26]. Another observational study including 90 adult patients (ELT, n = 58; ROM, n = 32) with ITP also reported that the response rate (*RR* = 0.83, 95%*CI*: 0.62–1.10), the proportion of patients discontinuing the treatment due to adverse reaction (*RR* = 0.83, 95%*CI*: 0.38–1.81), the incidence of any AEs (*RR* = 0.95, 95%*CI*: 0.52–1.74) and thrombosis (*RR* = 3.92, 95%*CI*: 0.21–73.50) were not significantly different between ELT and ROM [27]. Our results were consistent with these findings.

Nonetheless, an updated systematic review and meta-analysis of RCTs suggested that TPO-RAs were associated with a higher risk of thromboembolic events compared with placebo or standard care [28]. And another meta-analysis of three large, population-based observational studies concluded that the risk of arterial and venous thromboembolism should be considered when evaluating the risk of thromboembolism attributed to ITP treatments (e.g. TPO-RAs) [29]. Moreover, a disproportionality analysis in the World Health Organization global individual case safety report (ICSR) database (VigiBase) suggested the presence of a signal for an increased risk of thrombosis with ELT compared to ROM (adjusted reporting odds ratio = 1.72, 95%CI 1.47–2.02) [30]. However, our study did not find there was a significant difference, probably due to the small sample size. The physicians should still be cautious when administering TPO-RAs to patients with higher risk of thromboembolism, especially for ELT.

Long-term observational studies (2 for ROM [31][32] and 4 for ELT [33–36], respectively) indicated that a minority of patients treated with ROM or ELT developped bone marrow fibrosis and those adverse events (3 for ROM [37–39] and 1 for ELT [40], respectively) were usually reversible and dose dependent. Probably due to short-term follow-up and small sample size of the RCTs included in this review, the reticulin fibrosis in bone marrow was only reported in the ROM trials. But careful monitoring is still needed during use of ROM or ELT.

There are several limitations in this study. We only included RCTs in this review, the results might not have good generalizability for strict inclusion criteria and small sample size in those studies. These studies were not sensitive to find rare AEs (e.g. reticulin fibrosis in bone marrow) related to the drug as the sample size was relatively small. In addition, the results of indirect comparisons between ELT and ROM should be interpreted with caution due to lower power of test and heterogeneity caused by the study designs, patient populations, different dosage and course of treatment, and response definitions.

## Conclusions

In summary, this meta-analysis suggests that ELT and ROM might be similar in efficacy and safety for adult ITP. However, physicians should still take into account drug cost and comorbidities of the specific patient while making decisions on the treatment of ITP with TPO-RAs.

## Supporting information

S1 Table PRISMA 2009 checklist. (DOC)

S2 Table Searching strategy for PUBMED, EMBASE, and COCHRANE. (DOC)

## References

[1] Rodeghiero F, Stasi R, Gernsheimer T, Michel M, Provan D, Arnoid DM, et al Standardization of terminology, definitions and outcome criteria in an international working group immune thrombocytopenic purpura of adults and children: report from an international working group. Blood 2009;113:2386–2393. doi: 10.1182/blood-2008-07-162503 19005182 10.1182/blood-2008-07-162503

[2] Kurata Y, Fujimura K, Kuwana M, Tomiyama Y, Murata M. Epidemiology of primary immune thrombocytopenia in children and adults in Japan: a population-based study and literature review. Int J Hematol 2011;93:329–335. doi: 10.1007/s12185-011-0791-1 21347644 10.1007/s12185-011-0791-1

[3] Moulis G, Palmaro A, Montastruc JL, Godeau B, Lapeyre-Mestre M, Sailler L. Epidemiology of incident immune thrombocytopenia: a nationwide population-based study in France. Blood 2014;124:3308–3315. doi: 10.1182/blood-2014-05-578336 25305203 10.1182/blood-2014-05-578336

[4] Lee JY, Lee JH, Lee H, Kang B, Kim JW, Lee JO, et al Epidemiology and management of primary immune thrombocytopenia: A nationwide population-based study in Korea. Thromb Res 2017;155:86–91. doi: 10.1016/j.thromres.2017.05.010 28525829 10.1016/j.thromres.2017.05.010

[5] Terrell DR, Beebe LA, Neas BR, Vesely SK, Segal JB, George JN. Prevalence of primary immune thrombocytopenia in Oklahoma. Am J Hematol 2012;87:848–852. doi: 10.1002/ajh.23262 22674643 10.1002/ajh.23262 PMC3429719

[6] Neunert C, Lim W, Crowther M, Cohen A, Solberg L Jr, Crowther MA, et al The American Society of Hematology 2011 evidence-based practice guideline for immune thrombocytopenia. Blood 2011; 117:4190–4207. doi: 10.1182/blood-2010-08-302984 21325604 10.1182/blood-2010-08-302984

[7] Wang L, Gao Z, Chen XP, Zhang HY, Yang N, Wang FY, et al Efficacy and safety of thrombopoietin receptor agonists in patients with primary immune thrombocytopenia: a systematic review and meta-analysis. Sci Rep-UK 2016;6:39003. 10.1038/srep39003 PMC5171907 27991534

[8] Imbach P, Crowther M. Thrombopoietin-receptor agonists for primary immune thrombocytopenia. N Engl J Med 2011;365:734–741. doi: 10.1056/NEJMct1014202 21864167 10.1056/NEJMct1014202

[9] Erhardt JA, Erickson-Miller CL, Aivado M, Abbound M, Pillarisetti K, Toomey JR. Comparative analyses of the small molecule thrombopoietin receptor agonist eltrombopag and thrombopoietin on in vitro platelet function. Exp Hematol 2009;37:1030–1037. doi: 10.1016/j.exphem.2009.06.011 19631713 10.1016/j.exphem.2009.06.011

[10] Jansen JP, Fleurence R, Devine B, Itzler R, Barrett A, Hawkins N, et al Interpreting indirect treatment comparisons and network meta-analysis for health-care decision making: Report of the ISPOR Task Force on Indirect Treatment Comparisons Good Research Practices: Part 1. Value in Health 2011;14:417–428. doi: 10.1016/j.jval.2011.04.002 21669366 10.1016/j.jval.2011.04.002

[11] National Institute for Health and Clinical Excellence (NICE). Guide to the methods of technology appraisal. Available at: www.nice.org.uk/aboutnice/howwework/devnicetech/guidetothemethodsoftechnologyappraisal.jsp.2008. 27905712

[12] Cooper KL, Dillingham K, Helme K, Fitzgerald P, Akehurst R. Romiplostim and Eltrombopag for immune thrombocytopenia: methods for indirect comparison. Int J Technol Assess 2012;28:249–258. 10.1017/S0266462312000414 22980701

[13] Higgins J, Green S. The Cochrane Collaboration.Cochrane handbook for systematic reviews of interventions version 5.1.0. 2011. Available at: http://handbook.cochrane.org/.

[14] Higgins JP, Thompson SG. Quantifying heterogeneity in a meta-analysis. Stat Med 2002;21:1539–1558. doi: 10.1002/sim.1186 12111919 10.1002/sim.1186

[15] Li S, Zhang C, Yuan RX, Xu C, Zeng XT. Brief introction of indirect comparison software. Clin J Evid-based Med 2015;15:362–366.

[16] Bussel JB, Cheng G, Saleh MN, Psaila B, Kovaleva L, Meddeb B, et al Eltrombopag for the Treatment of Chronic Idiopathic Thrombocytopenic Purpura. N Engl J Med 2007;357:2237–2247. doi: 10.1056/NEJMoa073275 18046028 10.1056/NEJMoa073275

[17] Bussel JB, Provan D, Shamsi T, Cheng G, Psaila B, Kovaleva L, et al Effect of eltrombopag on platelet counts and bleeding during treatment of chronic idiopathic thrombocytopenic purpura: a randomized, double-blind, placebo-controlled trial. Lancet 2009;373:641–648. doi: 10.1016/S0140-6736(09)60402-5 19231632 10.1016/S0140-6736(09)60402-5

[18] Cheng G, Saleh MN, Marcher C, Vasey S, Mayer B, Aivado M, et al Eltrombopag for management of chronic immune thrombocytopenia (RAISE): a 6-month, randomized, phase 3 study. Lancet 2011;377:393 doi: 10.1016/S0140-6736(10)60959-2 20739054 10.1016/S0140-6736(10)60959-2

[19] Tomiyama Y, Miyakawa Y, Okamoto S, Katsutani S, Kimura A, Okoshi Y, et al A lower starting dose of eltrombopag is efficacious in Japanese patients with previously treated chronic immune thrombocytopenia. J Thromb Haemost 2012;10:799–806. doi: 10.1111/j.1538-7836.2012.04695.x 22409309 10.1111/j.1538-7836.2012.04695.x

[20] Yang R, Li J, Jin J, Huang M, Yu Z, Xu X, et al Multicentre, randomized phase Ⅲ study of the efficacy and safety of eltrombopag in Chinese patients with chronic immune thrombocytopenia. Brit J Haematol 2016;176:101–110. 27734464 10.1111/bjh.14380

[21] Bussel JB, Kuter DJ, George JN, McMillan R, Aledort LM, Conkin GT, et al AMG 531, a Thrombopoiesis-Stimulating Protein, for chronic ITP. N Engl J Med 2006;355:1672–1681. doi: 10.1056/NEJMoa054626 17050891 10.1056/NEJMoa054626

[22] Kuter DJ, Bussel JB, Lyons RM, Pullarkat V, Gernsheimer TB, Senecal FM, et al Efficacy of romiplostim in patients with chronic immune thrombocytopenia purpura: a double-blind randomized controlled trial. Lancet 2008;371:395–403. doi: 10.1016/S0140-6736(08)60203-2 18242413 10.1016/S0140-6736(08)60203-2

[23] Shirasugi Y, Ando K, Miyazaki K, Tomiyama Y, Okamoto S, Kurokawa M, et al Romiplostim for the treatment of chronic immune thrombocytopenia in adult Japanese patients: a double-blind, randomized Phase III clinical trial. Int J Hematol 2011;94:71–80. doi: 10.1007/s12185-011-0886-8 21706145 10.1007/s12185-011-0886-8

[24] Cooper K, Matcham J, Helme K, Akehurst R. Update on romiplostim and eltrombopag indirect comparison. Int J Technol Assess 2014;30:129–130. 10.1017/S0266462313000767 24485057

[25] Kurter DJ, Macahilig C, Grotzinger KM, Poston SA, Wang PF, Dawson KL, et al Treatment patterns and clinical outcomes in patients with chronic immune thrombocytopenia(ITP) switched to eltrombopag or romiplostim. Int J Hematol 2015;101:255–263. doi: 10.1007/s12185-014-1731-7 25586660 10.1007/s12185-014-1731-7

[26] Mazza P, Minoia C, Melpignano A, Polimeno G, Cascavilla N, Di Renzon N, et al The use of thrombopoietin-receptor agonists(TPO-RAs) in immune thrombocytopenia(ITP): a “real life” retrospective multicenter experience of the Rete Ematologica Pugliese(REP). Ann Hematol 2016;95:239–244. doi: 10.1007/s00277-015-2556-z 26596973 10.1007/s00277-015-2556-z

[27] Depré F, Aboud N, Ringel F, Salama A. Thrombopoietin receptor agonists are often ineffective in immune thrombocytopenia and/or cause adverse reactions: results from one hand. Transfus Med Hemother 2016;43:375–379. doi: 10.1159/000446195 27781025 10.1159/000446195 PMC5073627

[28] Catalá-López F, Corrales I, Fuente-Honrubia CDL, González-Bermejo D, Martin-Serrano G, Montero D, et al Risk of thromboembolism with thrombopoietin receptor agnonists in adult patients with thrombocytopenia: An updated systematic review and meta-analysis of randomized controlled trials. Medical Clinica 2015;145:511. 10.1016/j.medcli.2015.03.014 26051432

[29] Langeberg WJ, Schoonen WM, Eisen M, Gamelin L, Stryker S. Thromboembolism in patients with immune thrombocytopenia(ITP): a meta-analysis of observational studies. Int J Hematol 2016;103:655–664. doi: 10.1007/s12185-016-1974-6 27084254 10.1007/s12185-016-1974-6

[30] Nguyen TT, Palmaro A, Montastruc F, Lapeyre-Mestre M, Moulis G. Signal for thrombosis with eltrombopag and romiplostim: a disproportionality analysis of spontaneous reports within Vigi Base. Drug Saf 2015;38:1179–1186. doi: 10.1007/s40264-015-0337-1 26338346 10.1007/s40264-015-0337-1

[31] Kuter DJ, Mufti G, Bain BJ, Hasserjian RP, Davis W, Rutstein M. Evaluation of bone marrow reticulin formation in chronic immune thrombocytopenia patients treated with romiplostim. Blood 2009;114:3748–3756. doi: 10.1182/blood-2009-05-224766 19671919 10.1182/blood-2009-05-224766

[32] Steurer M, Quittet P, Papadaki HA, Selleslag D, Viallard JF, Kaiafa G, et al A large observational study of patients with primary immune thrombocytopenia receiving romiplostim in European clinical practice. Eur J Haematol 2017;98:112–120. doi: 10.1111/ejh.12807 27557853 10.1111/ejh.12807

[33] Wong RSW, Saleh MN, Khelif A, Salama A, Protella MSO, Burgess P, et al Safety and efficacy of long-term treatment of chronic/persistent ITP with eltrombopag: final results of the EXTEND study. Blood 2017;130:2527–2536. doi: 10.1182/blood-2017-04-748707 29042367 10.1182/blood-2017-04-748707

[34] Brynes RK, Orazi A, Theodore D, Burgess P, Bailey CK, Thein MM, et al Evaluation of bone marrow reticulin in patients with chronic immune thrombocytopenia treated with eltrombopag: Data from the EXTEND study. Am J Hematol 2015;90:598–601. doi: 10.1002/ajh.24011 25801698 10.1002/ajh.24011

[35] Gonzalez-Lopez TJ, Fernandez-Fuertes F, Hernandez-Rivas JA, Sanchez-Gonzalez B, Martinez-Robles V, Alvare-Roman MT, et al Efficacy and safety of eltrombopag in persistent and newly diagnosed ITP in clinical practice. Int J Hematol 2017;106:508–516. doi: 10.1007/s12185-017-2275-4 28667351 10.1007/s12185-017-2275-4

[36] Kim YK, Lee SS, Jeong SH, Ahn JS, Yang DH, Lee JJ, et al Efficacy and safety of eltrombopag in adult refractory immune thrombocytopenia. Blood Res 2015;50:19–25. doi: 10.5045/br.2015.50.1.19 25830126 10.5045/br.2015.50.1.19 PMC4377333

[37] Rashidi A and Roullet MR. Romiplostim-induced myelofibrosis. Blood 2013;122:2001 24175353 10.1182/blood-2013-05-500157

[38] Kirito K and Komatsu N. Progression of bone marrow fibrosis with reticulin and collagen hyperplasia during treatment with the thrombopoietin receptor agonist romiplostim in a patient with immune thrombocytopenia. Rinsho Ketsueki 2013;54:295–299. 23676646

[39] Gonzalez MF and Freeman JK. Myelofibrosis associated with romiplostim treatment in a patient with immune thrombocytopenia. Case Rep Hematol 2012;2012:318597 doi: 10.1155/2012/318597 22937324 10.1155/2012/318597 PMC3420420

[40] Horikoshi A, Tsukuda J, Abe R, Fujiwara N, Ito E, Takaku T. Development of myelofibrosis during eltrombopag treatment in a patient with immune thrombocytopenia. Rinsho Ketsueki 2016;57:638–641. doi: 10.11406/rinketsu.57.638 27263792 10.11406/rinketsu.57.638

