# Supplementary material for: Eltrombopag versus romiplostim in treatment of adult patients with immune thrombocytopenia: A systematic review incorporating an indirect-comparison meta-analysis
Source: PLoS One. 2018 Jun 1;13(6):e0198504. doi: 10.1371/journal.pone.0198504 (PMC5983520; doi:10.1371/journal.pone.0198504)
Supplement: S2 Table — (DOC) [file pone.0198504.s002.doc]

**S2 Table.** Searching Strategy

| **Database** | **Search Strategy** | **Results** |
| --- | --- | --- |
| PUBMED | **#**1 Thrombopoietin [Mesh] #2 Thrombopoietin* [Title/Abstract] #3 TPO [Title/Abstract]  #4 Eltrombopag [Supplementary Concept]   #5 Romiplostim [Supplementary Concept]  #6 Eltrombopag [Title/Abstract]  #7 Romiplostim [Title/Abstract]  #8 Nplate [Title/Abstract]  #9 Promacta [Title/Abstract]  #10 Revolade [Title/Abstract]  #11 **#**1~#10 OR  #12 "Purpura, Thrombocytopenic, Idiopathic"[MESH]  #13 Thrombocytopeni* [Title/Abstract]  #14 ITP  # 15 #12 OR #13 OR #14  #16 #11 and -#15 | 1654 |
| EMBASE | **#**1 Thrombopoietin [Emtree] #2 Thrombopoietin$ [Title/Abstract] #3 TPO [Title/Abstract]  #4 Eltrombopag [Emtree]   #5 Romiplostim [Emtree]  #6 Eltrombopag [Title/Abstract]  #7 Romiplostim [Title/Abstract]  #8 Nplate [Title/Abstract]  #9 Promacta [Title/Abstract]  #10 Revolade [Title/Abstract]  #11 **#**1~#10 OR  #12 "Purpura, Thrombocytopenic, Idiopathic"[Emtree]  #13 Thrombocytopeni$ [Title/Abstract]  #14 ITP [Title/Abstract]  # 15 #12 OR #13 OR #14  #16 #11 and -#15 | 1585 |
| COCHRANE | **#**1 Thrombopoietin [Title/Abstract/Key word] #2 Thrombopoietin* [Title/Abstract/Key word] #3 TPO [Title/Abstract/Key word]  #6 Eltrombopag [Title/Abstract/Key word]  #7 Romiplostim [Title/Abstract/Key word]  #8 Nplate [Title/Abstract/Key word]  #9 Promacta [Title/Abstract/Key word]  #10 Revolade [Title/Abstract/Key word]  #11 **#**1~#10 OR  #12 "Purpura, Thrombocytopenic, Idiopathic" [Title/Abstract/Key word]  #13 Thrombocytopeni* [Title/Abstract/Key word]  #14 ITP [Title/Abstract/Key word]  # 15 #12 OR #13 OR #14  #16 #11 and -#15 | 260 |
